# Image dataset of Taro Leaf Blight disease collected from the West African Sub-Region

**DOI:** 10.1016/j.dib.2025.111869

**Published:** 2025-07-10

**Authors:** Chidiebere Nwaneto, Chika Yinka-Banjo, Ogban-Asuquo Ugot, Obiageli Umeugochukwu, Thompson Annor

**Affiliations:** a University of Lagos Faculty of Science, Akoka, Lagos State, Nigeria; b Carl von Ossietzky Universitat Oldenburg, Germany; c Kwame Nkrumah University of Science and Technology, Ghana

**Keywords:** Phytophthora colocasia, Machine learning, Plant disease early detection, Deep Learning, West Africa Agriculture

## Abstract

This dataset encompasses an extensive collection of 18,248 high-resolution JPEG images, documenting various stages of Taro Leaf Blight (TLB) infection in Taro plants across West Africa. TLB, primarily caused by the pathogen Phytophthora colocasiae, manifests through necrotic leaf spots, white sporangia bands, and orange droplets, severely impacting the agricultural output and economic stability of smallholder farmers in the region. The images represent a range of infection stages—early, mid, late, and healthy conditions—captured during the dry and early rainy seasons in Nigeria and Ghana using smartphones equipped with high-resolution cameras.

This dataset was carefully curated to help in the development and training of machine learning models for early and accurate detection of TLB, a crucial step towards effective disease management. By enabling the application of advanced diagnostics through technologies such as smartphone apps and AI-based analysis tools, this dataset not only aims to enhance the technological capabilities within agricultural sectors but also serves as a vital educational resource. Researchers and developers can utilize this dataset to create and refine models that diagnose plant diseases promptly, thereby allowing for timely interventions that can prevent widespread crop damage and subsequent economic losses.

Additionally, the dataset supports ongoing efforts to integrate artificial intelligence with traditional farming practices, offering a bridge between advanced technological solutions and accessible applications for resource-limited settings. The potential reuse of this dataset extends beyond disease identification; it encompasses agricultural research, educational purposes, and further development of automated systems for plant health monitoring, making it a cornerstone for future innovations in agricultural technology and management strategies.

Specifications Table

**Table:** 

Subject	Agriculture and Computer Science.
Specific subject area	Plant disease detection using image data
Type of data	Image
Data collection	The data were collected by teams in Nigeria and Ghana, capturing different stages of TLB infection using smart phone cameras under natural field conditions within the dry and early rainy season [1].
Data source location	• **Institution**: University of Lagos, Akoka. Kwame Nkrumah University of Science and Technology • **City/Town/Region:** Abakaliki, Ebonyi, Izzi, Ezza North, Agbani, Ngwo, Ashanti. • **Country**: Nigeria and Ghana
Data accessibility	Repository name: Mendeley Data Data identification number: 10.17632/3knm93dkc5.1 Direct URL to data: https://data.mendeley.com/datasets/3knm93dkc5/1
Related research article	Nwaneto, C., Yiinka-Banjo, C., Ugot, O. A., Annor, T., & Umeugochukwu, O. (2024). EARLY DETECTION OF THE TARO LEAF BLIGHT DISEASE IN THE WEST AFRICAN SUB-REGION USING DEEP IMAGE CLASSIFICATION MODELS. *Smart Agricultural Technology*, 100,636.

## 1 Value of the Data

• This dataset is important for developing AI-driven tools for early TLB detection, which can significantly reduce crop losses and improve food availability for consumption.
• Researchers can utilize this dataset for training and validating machine learning models for early disease detection, particularly convolutional neural networks (CNNs), transfer learning frameworks like MobileNet, and real-time object detection models such as YOLO.
• The images are also valuable for educational purposes, teaching about plant health and disease progression.
• It provides critical support for AI-driven agricultural innovations in under-resourced regions.
• The dataset can aid in developing mobile diagnostic apps for farmers and extension workers.
• Educators and researchers in plant pathology and computer vision can use this dataset for academic purposes and cross-disciplinary training.
• Public access to metadata enhances reusability, allowing integration into broader datasets and future annotation studies.

## 2 Background

The need to enhance Taro crop yields in West Africa, in the midst of threats from Taro Leaf Blight (TLB), was the motivation that created this comprehensive image dataset [[2], [3]]. TLB, caused by Phytophthora Colocasiae, leads to significant yield losses, affecting the livelihoods of smallholder farmers. The disease thrives in warm, wet conditions typical of the West African climate, spreading rapidly through rain splash and wind, which makes early detection vital [4].

Recognizing the limitations faced by local farmers, including the high cost of advanced diagnostic technologies and a lack of technical expertise, this dataset aims to democratize access to effective disease management tools. By providing a robust dataset for training deep learning models, this initiative seeks to empower the development of accessible, mobile-based diagnostic applications. These tools can significantly mitigate the impact of TLB by enabling early detection and timely intervention, thus preserving crop health and farmer income. The data were collected from Taro fields in Nigeria and Ghana, capturing a broad spectrum of disease stages under natural growing conditions to ensure practical relevance and applicability.

## 3 Data Description

This dataset comprises 18,248 high-resolution JPEG images, each with dimensions of 500 × 500 pixels, documenting various stages of Taro Leaf Blight (TLB) infection. The images are organized into five distinct categories representing different stages of the disease progression as well as healthy plants, specifically designed to support the training and validation of machine learning models for disease detection. These categories are:

• Taro Early Blight (4864 images): Showcases the initial stages of the infection, characterized by smaller spots and less severe leaf damage.
• Taro Mid Blight (3370 images): Captures more advanced stages of the disease with more pronounced leaf spotting.
• Taro Late Blight (1270 images): Features leaves with severe damage, extensive spotting, and signs of leaf decay.
• Taro Healthy (8744 images): Includes images of healthy Taro leaves, which are crucial for models to learn to distinguish between infected and non-infected leaves.
• Taro Not-Early (4640 images): A combination of Mid and Late Blight stages, providing additional data for models to understand progression between these stages.

Each image file is labelled and structured within the dataset to ensure ease of access and use in model training scenarios. The uniformity in image size and quality facilitates the application of various image processing techniques without the need for pre-processing steps, making this dataset a ready-to-use resource for developing and testing deep learning algorithms aimed at managing and controlling TLB.

## 4 Experimental Design, Materials and Methods

The dataset was compiled to create a resource for the development of AI-driven diagnostics for Taro Leaf Blight (TLB). A collaborative effort between research teams in Nigeria and Ghana facilitated the collection of 18,248 high-resolution images, capturing diverse stages of TLB infection—Taro Early Blight, Taro Mid Blight, Taro Late Blight, and Taro Healthy. Each image, processed and standardized at 500 × 500 pixels in JPEG format, was captured using smartphones to reflect the real-world application and accessibility for local farmers [1].

Images were captured using a Xiaomi Redmi 10A smartphone equipped with an 8MP rear camera. The Xiaomi Redmi 10A smartphone was chosen as a representative low-end Android device commonly used in low-resource agricultural contexts. Photographs were taken at a close range of approximately 20–30 cm from the leaf surface, under natural daylight conditions during the day, with efforts to avoid shadows and maintain consistent angles for optimal image clarity.

Data collection occurred during both the dry and early rainy seasons to ensure variability in environmental conditions, which influence disease presentation. The images were taken in natural field settings, with careful consideration to maintain consistent lighting and angles to maximise the utility of the dataset for machine learning purposes, gathered from five agricultural zones in Nigeria and Ghana through several separate field visits.

A detailed data collection guideline was followed by the field team, which provided clear, consistent instructions for categorising each image under one of the four disease stage labels, such as ``Taro Early Blight,'' ``Taro Mid Blight,'' ``Taro Late Blight,'' and ``Taro Healthy.'' The collected images were then uploaded to a shared Google Drive folder.

A team of three plant pathology experts reviewed the images and conducted cross-validation to ensure labelling accuracy. Any misclassified or poor-quality images were excluded during a visual inspection-based quality control process.

During image processing, all images were resized to 500 × 500 pixels to ensure uniformity, preserve relevant visual features, and improve computational efficiency for deep learning model training and inference. Due to field constraints and the nature of mobile data collection, we were unable to annotate all files with detailed metadata (e.g., timestamp) at the point of capture. However, core categories and quality standards were applied consistently across the dataset.

## 5 Materials

• Low-end Android smartphone cameras.
• Google Drive folder.
• Data collection guideline document.

## 6 Justification for 500 × 500 pixel resolution

Resizing to 500 × 500 allows for retaining a reasonable amount of visual information necessary for the models to discern key features of the Taro Leaf Blight at different stages [5].

Computational efficiency: Using a uniform resolution helps in creating consistent input sizes for the models, which can streamline processing and reduce computational overhead during training and inference [6].

## Limitations

The dataset may not cover all possible environmental conditions under which TLB manifests, which could affect model generalization.

## Ethics Statement

This research complies with ethical standards for research without involving human or animal subjects.

## CRediT Author Statement

**Chidiebere B. Nwaneto:** Conceptualization, Formal analysis, Funding acquisition, Investigation, Methodology, Resources, Validation, Visualization, Writing – original draft, Writing – editing. **Ogban-Asuquo Ugot:** Conceptualization, Investigation, Methodology, Project administration, Resources, Software, Supervision, Validation, Writing – review & editing. **Chika Yinka-Banjo:** Conceptualization, Investigation, Methodology, Project administration, Resources, Supervision, Validation, Visualization, Writing – review. **Obiageli Umeugochukwu:** Conceptualization, Data Curation. **Thompson Annor:** Data Curation.

## Data Availability

Mendeley DataAn Image Dataset of Taro Leaf Blight Disease Collected from the West African Sub-Region (Original data).
